# Appropriate strategies for South Africa for the management of acute myocardial infarction in patients presenting with ST-segment elevation

**Published:** 2018

**Authors:** Delport Rhena

**Affiliations:** ST-Elevation Myocardial Infarction South Africa: National Project Manager, SA Heart Association; Department of Chemical Pathology, University of Pretoria, Pretoria, South Africa

## Timely reperfusion

Both patient and health-system delays contribute to delays inrestoring myocardial perfusion. Patient-related delays constitutethe time from onset of symptoms to the call for help, and onsetof symptoms to first medical contact. These will not be discussedhere. Of relevance are time metrics relating to ‘symptom onset toinitiation of fibrinolysis or first balloon or device, and hospitaldoor to either onset of fibrinolytic therapy (door-to-needle time)or to first balloon or device (door-to-balloon time)’, as well asstrategies that may be considered if circumstances are not ideal,as elegantly interrogated by Gershlick *et al*.^1^

Evidence-based guidelines provide clear time targets and recommendations for reperfusion therapy, as discussed in the report by Ibanez et al.,^2^ but in essence, primary percutaneous coronary intervention (PCI) is advocated as the primary strategy, and if not anticipated to be achievable within 120 minutes of ST-elevation myocardial infarction (STEMI) diagnosis, fibrinolysis should be initiated immediately. The study by Stassen et al., published in this edition (page 6), reports on the feasibility of PCI within the proposed time frame, while considering driving times and distances to public and private PCI facilities in different regions of South Africa.

## Mortality data

In the 2015 list of the 10 leading underlying natural causes of death in each province, the Western Cape (WC) ranks third, with ischaemic heart diseases (ICD-10: I20-I25) as cause of death (5.8% of all-cause deaths), followed by Gauteng (GP) ranking seventh (3% of all-cause deaths), and KwaZulu-Natal (KZN) ranking ninth (2.6% of all-cause deaths).^3^ Despite 100% of the inhabitants of GP living within 120 minutes of a PCI facility, calculated from mid-year population estimates for 2015,4 the proportionate mortality rate of 0.22/1 000 was higher than the mortality rate of 0.19/1 000 for KZN and markedly lower than that of 0.47/1 000 for the WC region, the respective proportions of inhabitants living within 120 minutes of a PCI facility for the latter two regions being 64.7 and 87.6%. These findings suggest that factors other than proximity to PCI facilities explain ischaemic heart disease mortality rates for South Africa.

## Disparities

Statistics South Africa (2011)5 reports a total medical aid coverage of 16% for the total population, the respective population covered by medical aid or medical benefit schemes or other private health insurance, and for the three aforementioned regions it is 23.7% for GP, 12.2% for KZN and 25% for WC. Access to healthcare facilities includes means of transport to reach the health facility normally used (walking: 47.4%, public transport: 29.1%, or own transport: 22.1%) and time taken to reach the health facility normally used (80% of households take less than 30 minutes to reach the health facility normally used).

Information relating to type of health facility used first when household members fell ill and decided to seek medical help may be relevant when interpreting the report of Stassen et al. on the proportion of the South African population living within 60 and 120 minutes of a public PCI facility. The authors calculated that 32.6 million (63%) of the total population lived within 120 minutes of a public PCI facility, whereas in reality only 9.5% of the population purportedly used public hospitals and 2.0% used private hospitals when household members fell ill. Public sector services are sourced by 70.6% of the total population (mainly public clinic services: 61.2%) and private sector services by 27.9% (mainly private doctor services: 24.3%).

Coming back to PCI services in particular, Stassen et al. previously reported that 48 (77%) PCI facilities are privately owned, whereas the 14 state-owned facilities are tasked with providing services to the population with no medical aid coverage (79.9%) and a high poverty rate (59.6%).^6^

## Recommendations

Given all these disparities, compounded by population dispersion across metropolitan and rural regions, equitable healthcare related to PCI services may be deemed questionable for South Africa. The White Paper on Management of STEMI in Lowand Middle-Income Countries by Baliga et al. 7 provides muchneeded insight into the challenges experienced in countries such as South Africa, relating, among others, to lack of essential resources and services. Clear strategies are proposed, as in the contribution of Gershlick et al.,^1^ which may be recommended for the formulation of solutions to enable prevention or reduction in STEMI-related mortality and morbidity in South Africa.
